# Metabolomic analysis of hydroxycinnamic acid inhibition on *Saccharomyces cerevisiae*

**DOI:** 10.1007/s00253-023-12830-8

**Published:** 2024-01-22

**Authors:** Xiaoli Ge, Junxiao Chen, Jie Gu, Wenbo Yi, Shujie Xu, Liping Tan, Tongjun Liu

**Affiliations:** 1 State Key Laboratory of Biobased Material and Green Papermaking, Qilu University of Technology, Shandong Academy of Sciences, Jinan, 250353 China; 2 Department of Bioengineering, Qilu University of Technology, Shandong Academy of Sciences, Jinan, 250353 China

**Keywords:** *Saccharomyces cerevisiae*, Ferulic acid, *P*-coumaric acid, Inhibitor, Untargeted metabolomics

## Abstract

**Abstract:**

Ferulic acid (FA) and* p*-coumaric acid (*p*-CA) are hydroxycinnamic acid inhibitors that are mainly produced during the pretreatment of lignocellulose. To date, the inhibitory mechanism of hydroxycinnamic acid compounds on *Saccharomyces cerevisiae* has not been fully elucidated. In this study, liquid chromatography-mass spectrometry (LC–MS) and scanning electron microscopy (SEM) were used to investigate the changes in *S. cerevisiae* cells treated with FA and *p*-CA. In this experiment, the control group was denoted as group CK, the FA-treated group was denoted as group F, and the *p*-CA-treated group was denoted as group P. One hundred different metabolites in group F and group CK and 92 different metabolites in group P and group CK were selected and introduced to metaboanalyst, respectively. A total of 38 metabolic pathways were enriched in *S. cerevisiae* under FA stress, and 27 metabolic pathways were enriched in *S. cerevisiae* under *p*-CA stress as identified through Kyoto Encyclopaedia of Genes and Genomes (KEGG) analysis. The differential metabolites involved included *S*-adenosine methionine, L-arginine, and cysteine, which were significantly downregulated, and acetyl-CoA, L-glutamic acid, and L-threonine, which were significantly upregulated. Analysis of differential metabolic pathways showed that the differentially expressed metabolites were mainly related to amino acid metabolism, nucleotide metabolism, fatty acid degradation, and the tricarboxylic acid cycle (TCA). Under the stress of FA and *p*-CA, the metabolism of some amino acids was blocked, which disturbed the redox balance in the cells and destroyed the synthesis of most proteins, which was the main reason for the inhibition of yeast cell growth. This study provided a strong scientific reference to improve the durability of *S. cerevisiae* against hydroxycinnamic acid inhibitors.

**Key points:**

• *Morphological changes of S. cerevisiae cells under inhibitors stress were observed.*

• *Changes of the metabolites in S. cerevisiae cells were explored by metabolomics.*

• *One of the inhibitory effects on yeast is due to changes in the metabolic network.*

## Introduction

The exhaustion of fossil fuels and the limitation of natural gas require enormous effort to produce renewable energy from environmentally friendly and sustainable sources. Biomass fuel ethanol is a valid candidate for renewable and sustainable fuel manufacturing (Zabed et al. 2016; Ruangrit et al. 2021). The conversion of lignocellulosic feedstocks to fuel ethanol requires pretreatment, enzymatic hydrolysis, and fermentation (Deshavath et al. 2021). As an excellent industrial ethanol-producing organism (Sharma et al. 2022), *S. cerevisiae* has the advantages of a short growth period and high fermentation capacity (Choi et al. 2010). During alkaline pretreatment, a large number of inhibitors are released, which are mainly phenolic compounds and weak acids (Ko et al. 2015; Jönsson and Martín 2016). These inhibitors may reduce cellulase activity, decrease the conversion of fermentable sugars, or have some impact on the growth and metabolism of yeast cells (Liu et al. 2019). Among these toxic compounds, phenolic compounds are generally considered the most harmful because they can seriously inhibit the growth and metabolism of *S. cerevisiae* (Adeboye et al. 2014; Gu et al. 2019).

Metabolomics is an emerging discipline after genomics, transcriptomics, and proteomics (Li et al. 2018). It is based on systems biology theories. It uses high-resolution and sensitive detection and data processing to analyze the overall changes of low-molecular metabolites in an organism after external stimulation. Metabolomics has been widely used to detect the changes in key metabolites and metabolic reactions in the microbial stress response. Metabolites are the products of complex biochemical reactions in cells and are the actual participants in the stress response (Wang et al. 2023). It is believed that metabolomics can reflect cellular metabolism more directly than transcriptomic or proteomic data. In recent years, many researchers have combined the stress of *S. cerevisiae* in various environments with metabolomics. Zeng et al. (2022) used metabolomics to study the impact of formic acid treatment on the metabolites of *S. cerevisiae* cells. They found that formic acid can inhibit protein synthesis and cause oxidative stress, thereby inhibiting the growth of yeast cells. Wang et al. (2015) studied the inhibitory effects of acetic acid, phenol, and furfural on *S. cerevisiae* through metabolomics. They found that proline could act as a reactive oxygen species (ROS) scavenger to protect the strain from inhibitor damage. Bo et al. (2014) used metabolomics to study the inhibitory effect of lysine on *S. cerevisiae*. The results showed that lysine inhibited the growth of *S. cerevisiae* by inhibiting the glycolysis pathway and tricarboxylic acid cycle, which inhibited the central metabolic pathway.

Although metabolomic analysis has been used to reveal key metabolites and metabolic pathways involved in microbial stress tolerance, we do not yet understand what metabolites are produced and what stress responses are involved in the inhibition of hydroxycinnamic acid compounds against *S. cerevisiae*. There is a need to investigate the mechanism of *S. cerevisiae* inhibition by inhibitors in lignocellulosic hydrolysate. In this study, scanning electron microscopy (SEM) combined with untargeted metabolomics was used to systematically analyze the inhibitory effects of FA (ferulic acid) and* p*-CA (*p*-coumaric acid) on *S. cerevisiae* at the metabolic level, to provide a scientific evidence for the study of the regulatory mechanism of yeast tolerance.

## Materials and methods

### Materials

The *S. cerevisiae* strain used in this study was obtained by our laboratory from an active dried yeast product from Angel Yeast Company (Yichang, Hubei, China). Mass spectrometry acetonitrile was purchased from Fisher (Pittsburgh, USA). Ferulic acid (FA), *p*-coumaric acid *(p*-CA), and formic acid were purchased from Sigma (St. Louis, USA). Yeast extract, peptone, and agar were purchased from Auboxing Biotechnology Co., Ltd. (Beijing, China).

### Effects of ferulic acid and p-coumaric acid on S. cerevisiae growth and glucose utilization

Different amounts of FA and* p*-CA were added to the YPD medium, and the final concentrations were 0 g/L, 0.5 g/L, 1.0 g/L, 1.5 g/L, and 2.0 g/L, respectively. Yeast cells were inoculated at 3% inoculum into the medium and incubated in an incubator (Minquan MQD-B3NR, Shanghai, China) at 30 °C and 160 r/min. Samples were taken at 2 h, 4 h, 6 h, 8 h, 12 h, 15 h, 24 h, 36 h, and 48 h of fermentation, respectively, and the absorbance of OD_600_ was measured by ultraviolet spectrophotometer (Metash Instrument UV-5500PC, Shanghai, China). The glucose content was determined by high-performance liquid chromatography (Shimadzu LC-20A, Kyoto, Japan), and the glucose consumption rate under FA and *p*-CA stress at different concentrations was compared (Yang et al. 2021).

### Effects of ferulic acid and p-coumaric acid on yeast cell morphology

FA (1.5 g/L) and* p*-CA (2.0 g/L) were added to a YPD culture medium with yeast strains. After culturing at 30 °C and 160 r/min for 24 h, the yeast solution was centrifuged at 4 °C and 5000 r/min for 15 min. The cells were washed three times with 0.05 mol/L PBS buffer (NaCl, KCl, KH_2_PO_4_, Na_2_HPO_4_) at pH 7.4 and then fixed it at 4 °C with 2.5% glutaraldehyde in a refrigerator for 12 h. The cells were washed three times with PBS buffer eluted using tert-butanol with gradient concentrations (50%, 70%, 80%, 90%, 100%), and finally solidified at 4 °C with tert-butanol and freeze-dried. Dried yeast cells were gold-sprayed using an E-1010 ion sputterer (Hitachi, Kyoto, Japan), and SEM images were taken at 5000 × magnification with a Regulus 8220 SEM (Hitachi, Kyoto, Japan).

### Preparation of metabolites

The activated strains were inoculated into YPD medium supplemented with 1.5 g/L and 2.0 g/L FA and *p*-CA, respectively, and cultured at 30 °C, 160 r/min for 24 h. Samples were drawn and centrifuged at 4 °C and stored for further analysis. Six biological replicates were set up in this experiment; the control group was denoted as group CK (CK**-**1, CK**-**2, CK**-**3, CK**-**4, CK**-**5, CK**-**6), the FA-treated group was denoted as group F (F**-**1, F**-**2, F**-**3, F**-**4, F**-**5, F**-**6), and the *p*-CA-treated group was denoted as group P (P**-**1, P**-**2, P**-**3, P**-**4, P**-**5, P**-**6). One thousand microliters of acetonitrile was added to the samples, and the ultrasonic washing device (KQ5200DE, Jiangsu, China) was used for ice-bath ultrasonic disruption treatment for 30 min. The samples were centrifuged at 8800 × *g* and 4 °C for 15 min. Six hundred microliter supernatant was taken and dried by vacuum centrifugation and dyed at 37 °C. The residue was dissolved with 100 μL acetonitrile and centrifuged at 8800 g for 10 min at 4 °C, and 10 μL of the supernatant was applied for untargeted metabolic analysis with the ultra performance liquid chromatography mass spectrometry (UPLC-MS) (TripleTof5600 + , SCIEX, Framingham, MA, USA) system.

### LC–MS analysis

The instrument used in this experiment was an UPLC (Shimadzu LC-30, Kyoto, Japan) with a SCIEX 5600 mass spectrometer using a Waters XBridge C18 column (Waters, Milford, MA, USA) (150 × 3 mm, 2.5 µm), operating at 35 °C, and the rate of flow was 0.300 ml/min. 0.1% CH_3_COOH was the mobile phase A, and B was CH_3_CN. The elution data of the mobile phase is shown in Table 1.

**Table 1:** Elution procedure of mobile phase

Time (min)	Parameter
0	A: 95%, B: 5%
8	A: 50%, B: 50%
10	A: 50%, B: 50%
13	A: 5%, B: 95%
12	A: 95%, B: 5%
15	A: 95%, B: 5%

The conditions of mass spectrometry were as follows: Electrospray ionization (ESI) was used to detect in negative and positive ion modes. The ion source temperature was 450 °C (negative ions) and 500 °C (positive ions), respectively. The ion spray voltage floating was 4400 V (negative ions) and 5500 V (positive ions), the scanning range of time of flight mass spectrometry (TOF MS) was 100–1200 Da, the scanning range of product ion was 50–1000 Da, and the scanning time and interval of TOF MS are 0.2 s and 0.01 s, respectively. Mass spectrometry uses information-dependent acquisition in a highly sensitive mode. The declustering potential was ± 60 V, and the collision energy was 35 ± 15 eV.

### Screening and analysis of differential metabolites

In this study, the screening conditions for relevant differential metabolites were *P* < 0.05 and variable important in projection (VIP) > 1 (Xiong et al. 2021). The samples from group F and group P were analyzed by principal component analysis (PCA), partial least square-discriminant analysis (PLS-DA), and cluster heat map analysis, respectively, with comparison to the group CK. The pathway enrichment analysis was carried out for the screened differential metabolites. The analysis was performed with the MetPA database, which is part of Metaboanalyst (http://www.metaboanalyst.ca) and is primarily based on the Kyoto Encyclopaedia of Genes and Genomes (KEGG) (http://www.kegg.jp/) metabolic pathways. Using the concentration of metabolic pathways and topological analysis, the metabolic pathways that may be affected by biodisturbance were identified (Deng et al. 2020), and analyzed for the significant metabolic pathways.

### Statistical analysis

In this study, we used Analysis Base File Converter software to analyze the results. Multivariate statistical analysis was performed by PCA and PLS-DA, respectively. Differential metabolites are selected based on the following thresholds:* P* < 0.05 and VIP > 1. There was a statistically significant difference between the groups.

## Results

### Effects of ferulic acid and p-coumaric acid on S. cerevisiae growth and glucose utilization

**Fig. 1:**
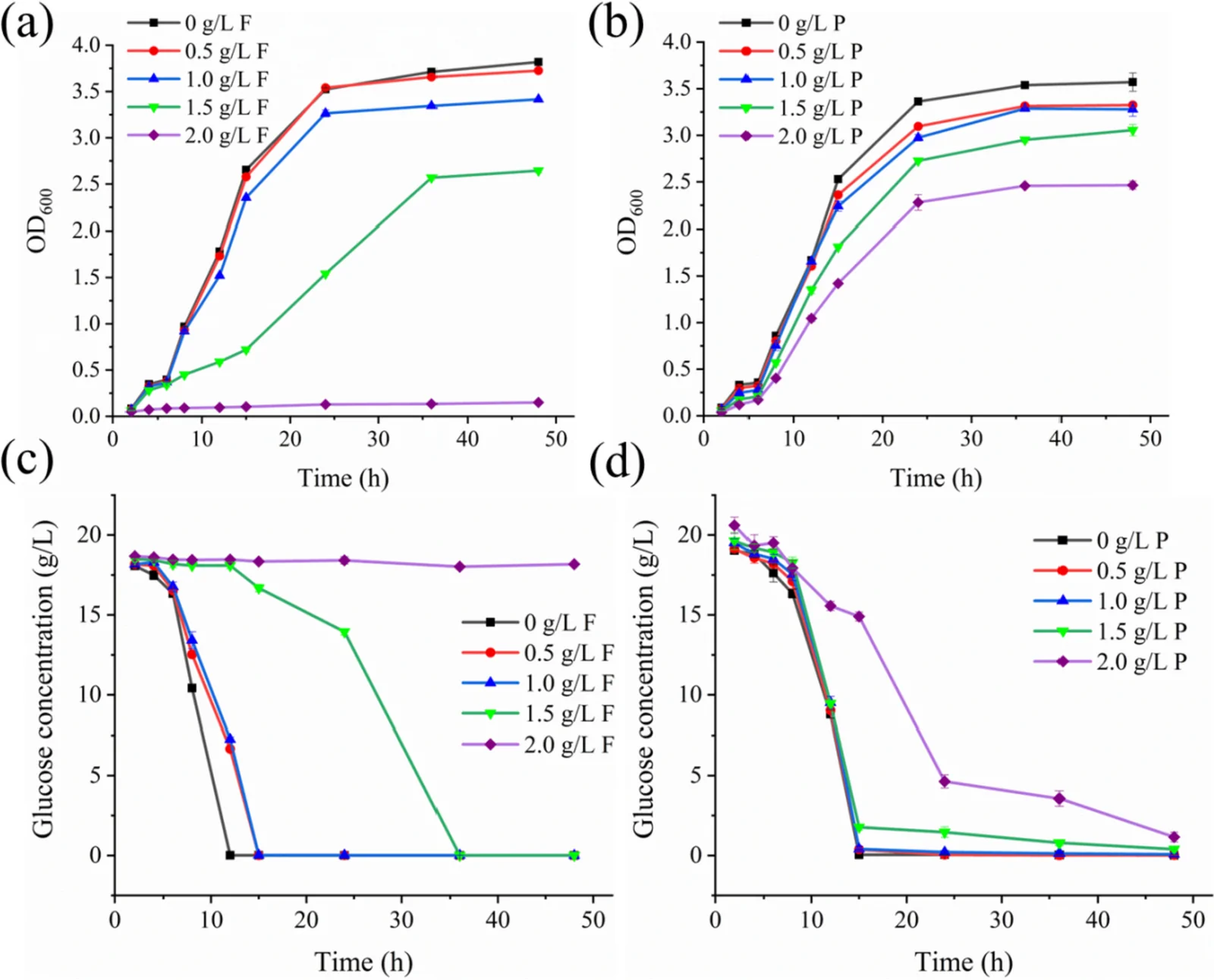
Effects of ferulic acid and* p*-coumaric acid on *S. cerevisiae* growth and glucose utilization. **a** Effect of ferulic acid on *S. cerevisiae* growth. **b** Effect of* p*-coumaric acid on *S. cerevisiae* growth. **c** Effect of ferulic acid on glucose utilization in *S. cerevisiae*. **d** Effects of *p*-coumaric acid on glucose utilization in *S. cerevisiae*

The effects of FA and* p*-CA on *S. cerevisiae* growth are shown in Fig. 1a, b. The inhibition of *S. cerevisiae* growth was not pronounced when FA and* p*-CA were added in low amounts. When FA was added with a concentration of 1.5 g/L, the OD_600_ decreased significantly, and the *S. cerevisiae* growth was seriously inhibited. When the concentration reached 2.0 g/L, the *S. cerevisiae* was severely hampered and did not grow. Inhibition by *p*-CA was weaker than that by FA. When the concentration of* p*-CA was 2.0 g/L, the growth of *S. cerevisiae* was significantly inhibited, and the yeast cell mass was greatly reduced.

The effects of FA and* p*-CA on glucose utilization in *S. cerevisiae* are shown in Fig. 1c, d. The glucose consumption was very different for different amounts of FA addition. When the FA concentration was 0–1.0 g/L, the glucose consumption did not change significantly, indicating that the low concentration of FA had an inhibitory effect on *S. cerevisiae* but did not affect its utilization of glucose. However, when the concentration was added to 1.5 g/L, the glucose consumption decreased significantly, and the residual glucose concentration decreased rapidly at 15 h, which showed that the *S. cerevisiae* had a high tolerance to 1.5 g/L FA. Under this concentration, the *S. cerevisiae* growth was seriously inhibited, the lag phase was prolonged, and the glucose consumption decreased. When the FA concentration was added to 2.0 g/L, the glucose consumption was very minimal, and it was evident that the *S. cerevisiae* growth was strongly inhibited. When the *p*-CA concentration was 0–1.5 g/L, the glucose consumption was slightly inhibited, but the inhibitory effect was not apparent. When the *p*-CA concentration reached 2.0 g/L, the glucose consumption decreased significantly. It began to decline rapidly at 15 h, indicating that the growth of *S. cerevisiae* was strongly inhibited under this condition.

Compared with FA, the glucose consumption of *p*-CA was faster under the same concentration. In addition, the former showed significant inhibition at 1.5 g/L, while the latter showed considerable inhibition only at 2.0 g/L. It indicated that FA has a more substantial inhibitory effect on *S. cerevisiae* than* p*-CA, which was consistent with the findings of *S. cerevisiae* growth experiments in this study.

### Effects of ferulic acid and p-coumaric acid on yeast cell morphology

**Fig. 2:**
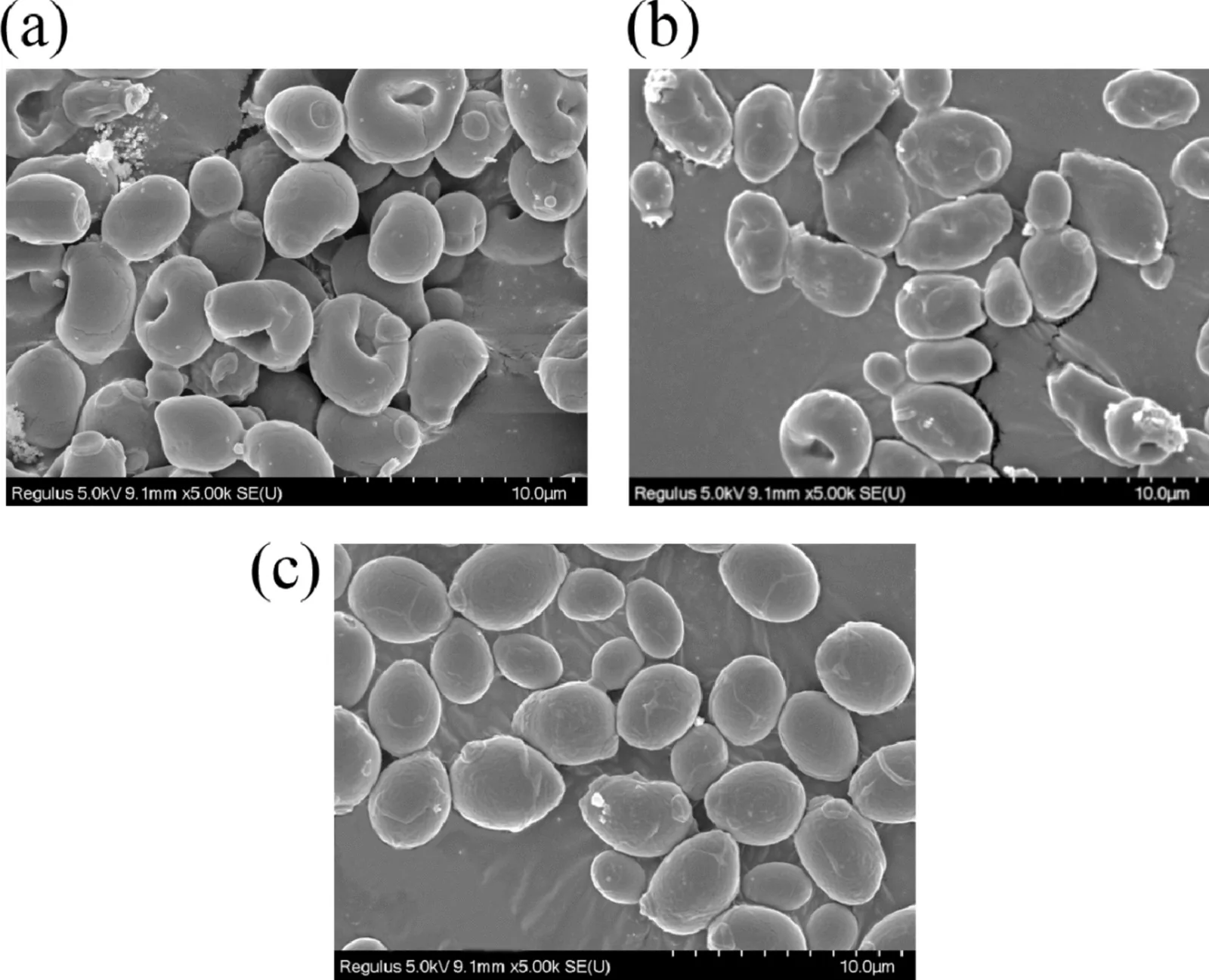
Scanning electron microscopy of yeast cells. **a** Yeast cells treated with 1.5 g/L ferulic acid. **b** Yeast cells treated with 2.0 g/L *p*-coumaric acid. **c** Yeast cells without inhibitor treatment

SEM images are shown in Fig. 2. Yeast cells without inhibitor treatment (Fig. 2c) had well-defined shapes (round or oval) and were round and full, with smooth cell surfaces without wrinkles. Yeast cells treated with 1.5 g/L FA (Fig. 2a) showed cell deformation, uneven cell surface, and noticeable cell shrinkage, indicating that adding FA had affected the expected growth of yeast cells. The yeast cells treated with 2.0 g/L* p*-CA (Fig. 2b) were deformed. The cell surface became rough, and the cells wrinkled. It is speculated that the addition of inhibitors changed the yeast cell morphology and affected the normal function of cell membranes and cell walls, resulting in the inability of nutrients to enter the cells, and affecting the normal growth and metabolism of the cells, which led to cell damage and even death.

### Principal component analysis of metabolomics

**Fig. 3:**
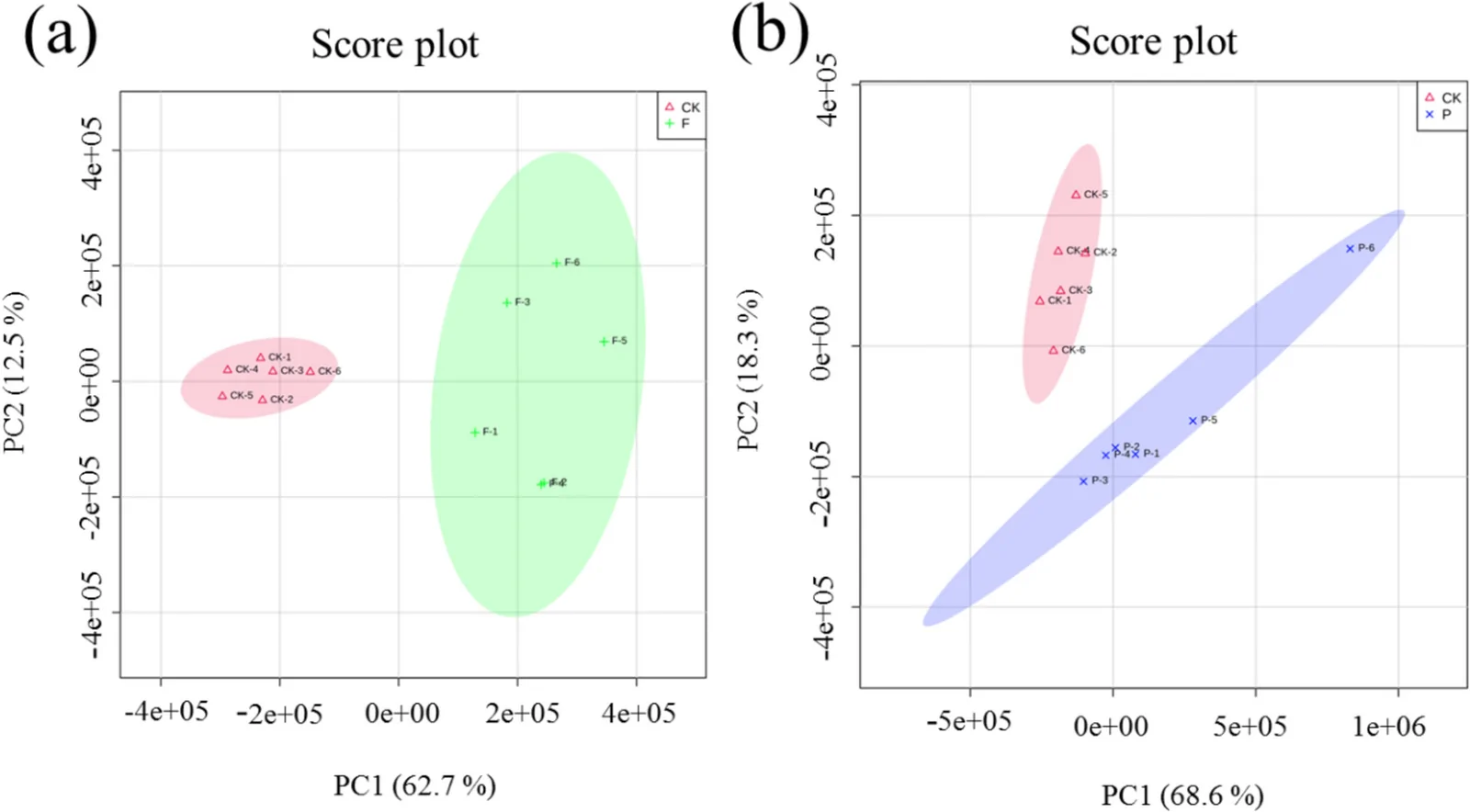
Principal component analysis scores of intracellular metabolites in the sample groups and the control group. Red represents group CK, green represents group F, and blue represents group P. **a** Principal component analysis score chart of the F and CK groups. **b** Principal component analysis score chart of the P and CK groups

To distinguish whether the group CK was different from the sample groups (F, P), PCA (Xiong et al. 2021) was used in this study. In Fig. 3a, group F and group CK samples were analyzed, adding the principal components of PC1 and PC2 to get the explanation rate of the model, which was 75.2%. Similarly, in Fig. 3b, the explanation rate of the model was 86.9%, within the 95% confidence interval. In the PCA score chart, the quality control (QC) samples were concentrated in the middle and almost overlapped, indicating that the whole analysis system was stable and reliable (Li et al. 2021). PCA results for both sample groups showed significant differences between the control and sample groups, indicating significant metabolic differences.

### Partial least square discriminant analysis

**Fig. 4:**
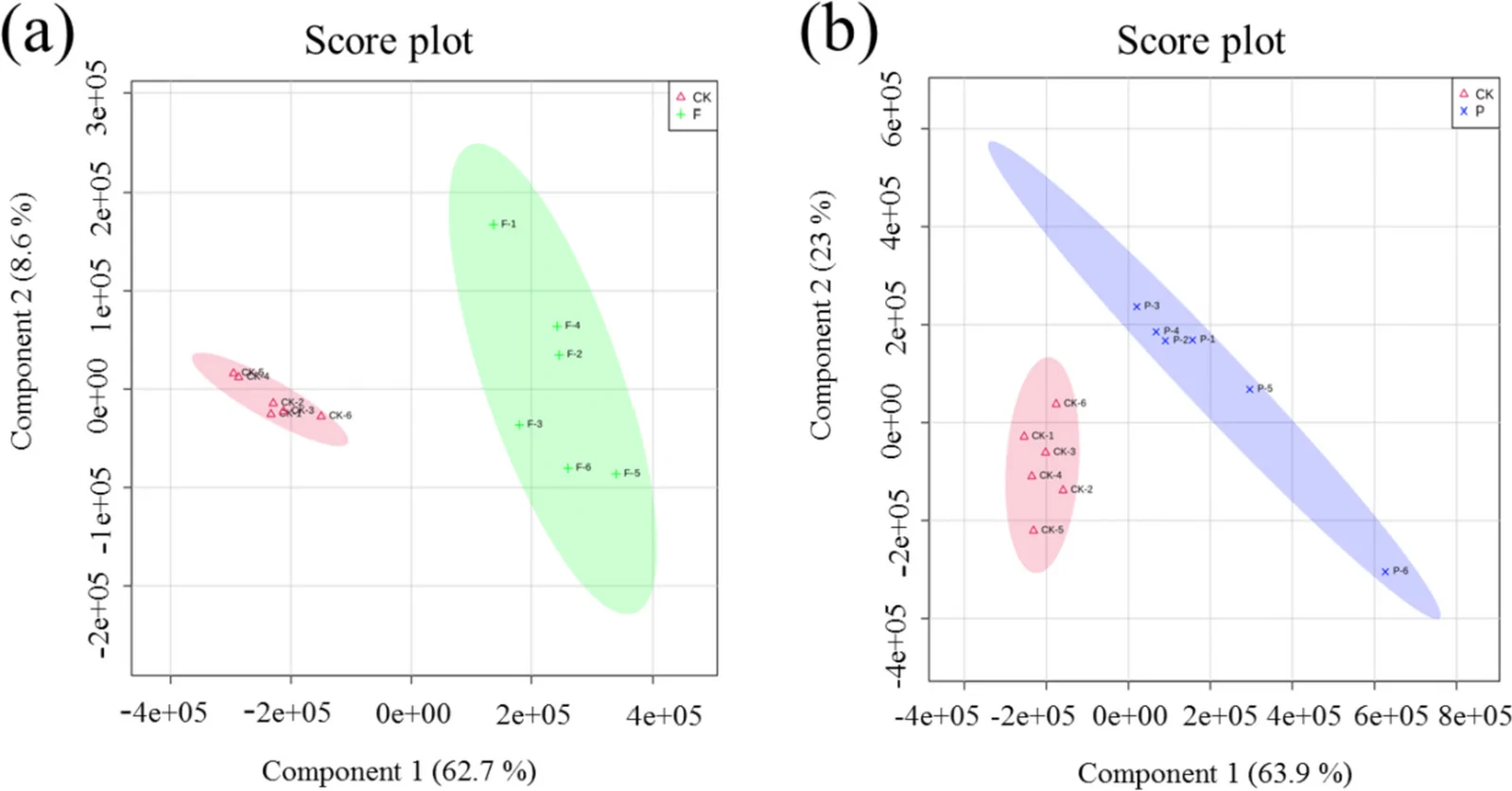
Partial least square discriminant analysis of intracellular metabolites in the sample groups and the control group. Red represents group CK, green represents group F, and blue represents group P. **a** Partial least square discriminant analysis score chart of the F and CK groups. **b** Partial least square discriminant analysis score chart of the P and CK groups

Unlike PCA analysis, PLS-DA analysis is another supervised analysis method (Li et al. 2021). The method designates and groups samples at the time of modeling, allowing greater identification of differences between groups. The PLS-DA score plot in Fig. 4 shows the sample differences between groups. The group CK and the sample groups were clustered into one category, and both of the groups could be completely separated, which was in agreement with the PCA results. It indicated that the experiment had good reproducibility, and that method was stable. The quality of the PLS-DA model was determined by parameters *R*^2^*Y* and *Q*^2^. *R*^2^*Y* indicates the goodness of the fit, and *Q*^2^ indicates the predictability of the model. The closer *R*^2^ and *Q*^2^ are to 1, the more reliable and stable the model is (Xu et al. 2020). In Fig. 4a, R^2^ = 0.908, *Q*^2^ = 0.740, and in Fig. 4b, R^2^ = 0.980 and *Q*^2^ = 0.830, it can be seen that these indicators were close to 1; it shows that the PLS-DA model is more reliable stable and stable, and can better predict the difference between the metabolites of yeast cells in the control and sample groups.

### Screening of differential metabolites

The criteria of VIP value > 1 in the PLS-DA model and *P* value < 0.05 in the *t* test were used to screen the metabolites with a significant difference. A total of 11 differential metabolites significantly downregulated and 89 upregulated from the F and CK groups were screened. A total of 27 significantly downregulated metabolites and 65 upregulated metabolites were screened from the P and CK groups. Tables 2 and 3 showed the significant differential metabolites in yeast cells treated with FA and* p*-CA, respectively.

**Table 2:** Some significant differential metabolites in yeast cells treated with ferulic acid

Number	Metabolite name	Formula	VIP (F vs CK)	*P* value (F vs CK)	
1	*sn*-Glycero-3-phosphocholine	C_8_H_21_NO_6_P	13.93	1.48E − 08	
2	Sorbitol-6-phosphate	C_6_H_15_O_9_P	4.17	0.00021	
3	*S*-Adenosyl-methionine	C_15_H_22_N_6_O_5_S	3.71	0.00013	
4	5′-*S*-Methylthioadenosine	C_11_H_15_N_5_O_3_S	3.47	7.75E − 05	
5	Valine	C_5_H_11_NO_2_	3.44	7.94E − 05	
6	Ophthalmic acid	C_11_H_19_N_3_O_6_	3.14	3.37E − 05	
7	Palmitic acid	C_16_H_32_O_2_	2.87	6.88E − 05	
8	Glutathione	C_10_H_17_N_3_O_6_S	2.65	5.54E − 05	
9	Isoleucine	C_6_H_13_NO_2_	2.50	0.020	
10	*S*-Adenosyl-L-homocysteine	C_14_H_20_N_6_O_5_S	2.30	4.66E − 05	
11	L-Arginine	C_6_H_14_N_4_O_2_	2.30	0.0042	
12	Acetyl coenzyme A	C_23_H_38_N_7_O_17_P_3_S	2.17	0.00063	
13	Protoporphyrin IX	C_34_H_34_N_4_O_4_	1.60	1.4E − 06	
14	Nicotinamide	C_6_H_6_N_2_O	1.35	1.82E − 06	
15	L-Glutamine	C_5_H_10_N_2_O_3_	1.18	1.07E − 06	
16	Adenosine	C_10_H_13_N_5_O_4_	1.16	9.68E − 05	
17	3-Ureidopropionic acid	C_4_H_8_N_2_O_3_	1.14	0.024	
18	Pantothenate	C_9_H_17_NO_5_	1.05	0.00047	
19	Hypoxanthine	C_5_H_4_N_4_O	1.00	2.31E − 07

**Table 3:** Some significant differential metabolites in yeast cells treated with* p*-coumaric acid

Number	Metabolite name	Formula	VIP (P vs CK)	*P* value (P vs CK)
1	*sn*-Glycero-3-phosphocholine	C_8_H_21_NO_6_P	11.53	0.0023
2	Adenosine 3′-monophosphate	C_10_H_14_N_5_O_7_P	5.45	0.0010
3	*S*-Adenosyl-methionine	C_15_H_22_N_6_O_5_S	3.64	0.00036
4	L-Glutamic acid	C_5_H_9_NO_4_	3.43	0.00015
5	Ophthalmic acid	C_11_H_19_N_3_O_6_	3.23	6.9557E − 05
6	AMP	C_10_H_14_N_5_O_7_P	2.78	0.0021
7	L-Arginine	C_6_H_14_N_4_O_2_	2.22	0.011
8	Protoporphyrin IX	C_34_H_34_N_4_O_4_	1.67	0.015
9	NAD^+^	C_21_H_28_N_7_O_14_P_2_	1.60	0.0022
10	Adenosine	C_10_H_13_N_5_O_4_	1.27	0.0043
11	L-threonine	C_4_H_9_NO_3_	1.03	0.0010

### Metabolic pathway enrichment analysis

**Fig. 5:**
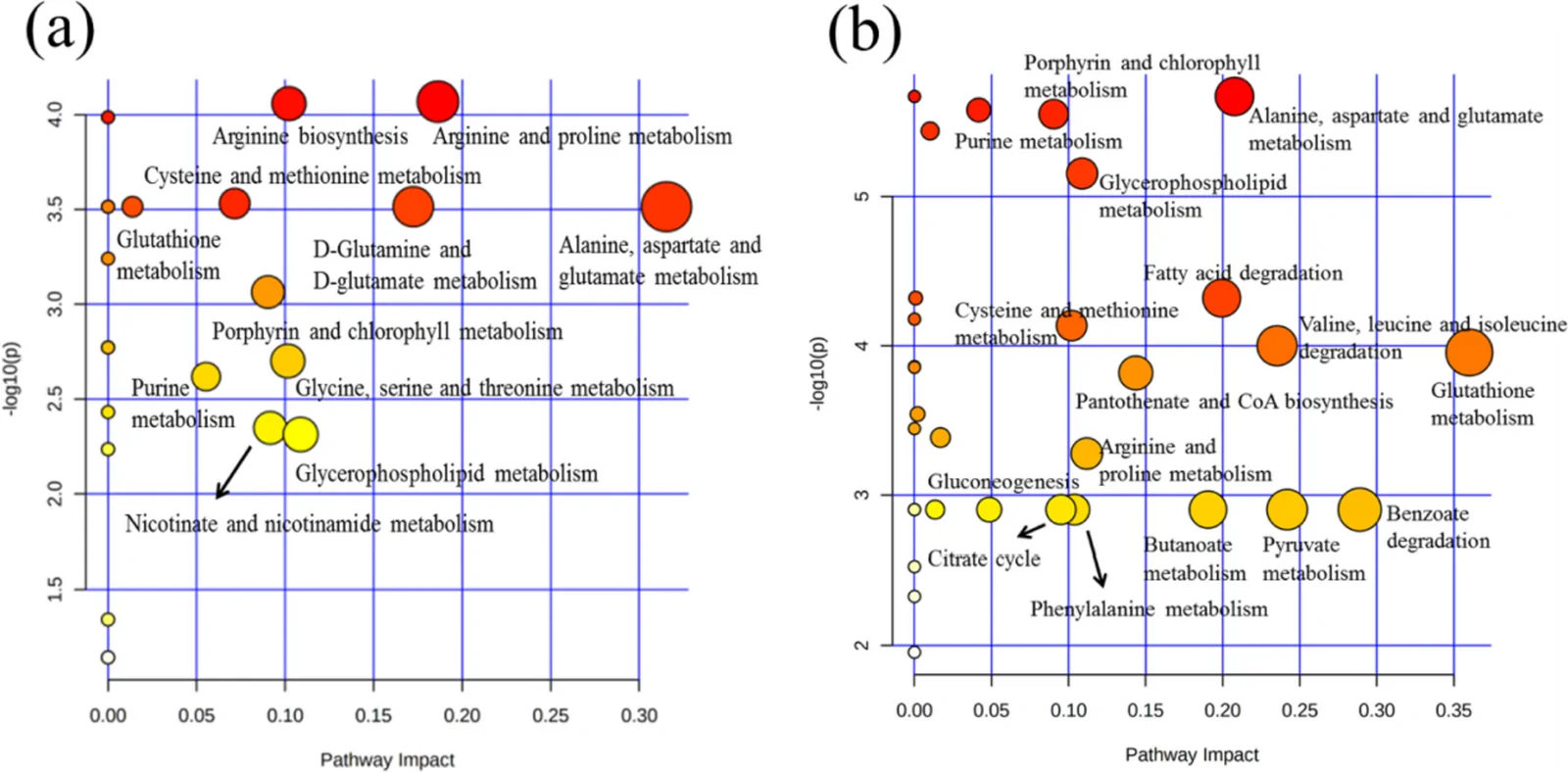
Analysis of metabolic pathways of significantly different metabolites. **a** Analysis of metabolite pathways with a significant difference under ferulic acid stress. **b** Analysis of metabolite pathways with a significant difference under *p*-coumaric acid stress

To further evaluate the changes in metabolic pathways in *S. cerevisiae* that occurred under FA and* p*-CA stress, we performed a KEGG pathway enrichment analysis on significantly differentially accumulated metabolites using the MetPA database (Sailwal et al. 2020), and identified the metabolic pathways that may be disturbed by bioturbation, and then, the metabolic pathways of these metabolites were analyzed. The 100 differential metabolites screened from group F and 92 differential metabolites screened from group P were each introduced into the MetaboAnalyst. KEGG pathway analysis showed 38 metabolic pathways enriched by *S. cerevisiae* under FA stress, of which 16 were significantly enriched (impact value > 0). There were 27 metabolic pathways enriched by *S. cerevisiae* under* p*-CA stress, of which 11 were significantly enriched. The metabolic pathway analysis is shown in Fig. 5. Each of these bubbles represents one KEGG pathway. The horizontal coordinate indicates the relative importance (impact value) of metabolites in the pathway, and the ordinate represents the enrichment significance-log10 (*P* value) of metabolite participation in the pathway. The impact value is represented by the bubble size. The pathway’s significance increases with the bubble size.

## Discussion

**Fig. 6:**
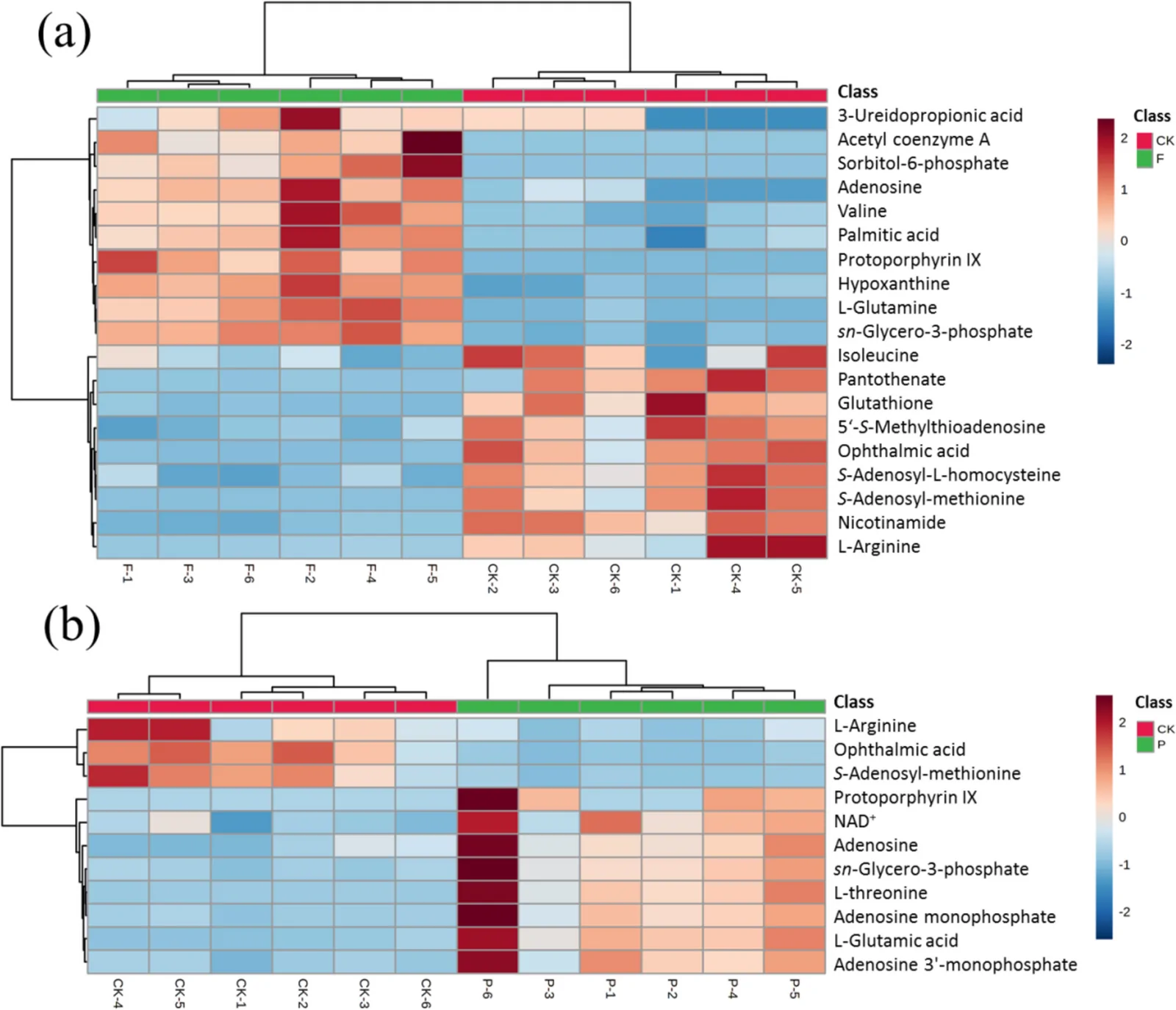
Cluster diagram of metabolite contents of ferulic acid and *p*-coumaric acid in yeast cells. The color of each section corresponds to the concentration of each metabolite. Red and blue represent upregulation and downregulation of metabolites, respectively. **a** Cluster diagram of significant differential metabolite contents in ferulic acid-treated yeast cells. **b** Cluster diagram of significant differential metabolite contents in *p*-coumaric acid-treated yeast cells

The effects of FA and* p*-CA on *S. cerevisiae* metabolism were investigated based on LC–MS metabolomics. The number and type of differential metabolites were significantly increased under the stress of FA and* p*-CA, which may be the result of the defense mechanism initiated by the cells upon stimulation (Xu et al. 2018). Analysis of metabolic pathways revealed that differentially expressed metabolites, as shown in Fig. 6, were mainly related to TCA cycle, amino acid metabolism, amino acid metabolism, nucleotide metabolism, and fatty acid degradation. The newly forming metabolic network could cope with FA and* p*-CA stress.

**Fig. 7:**
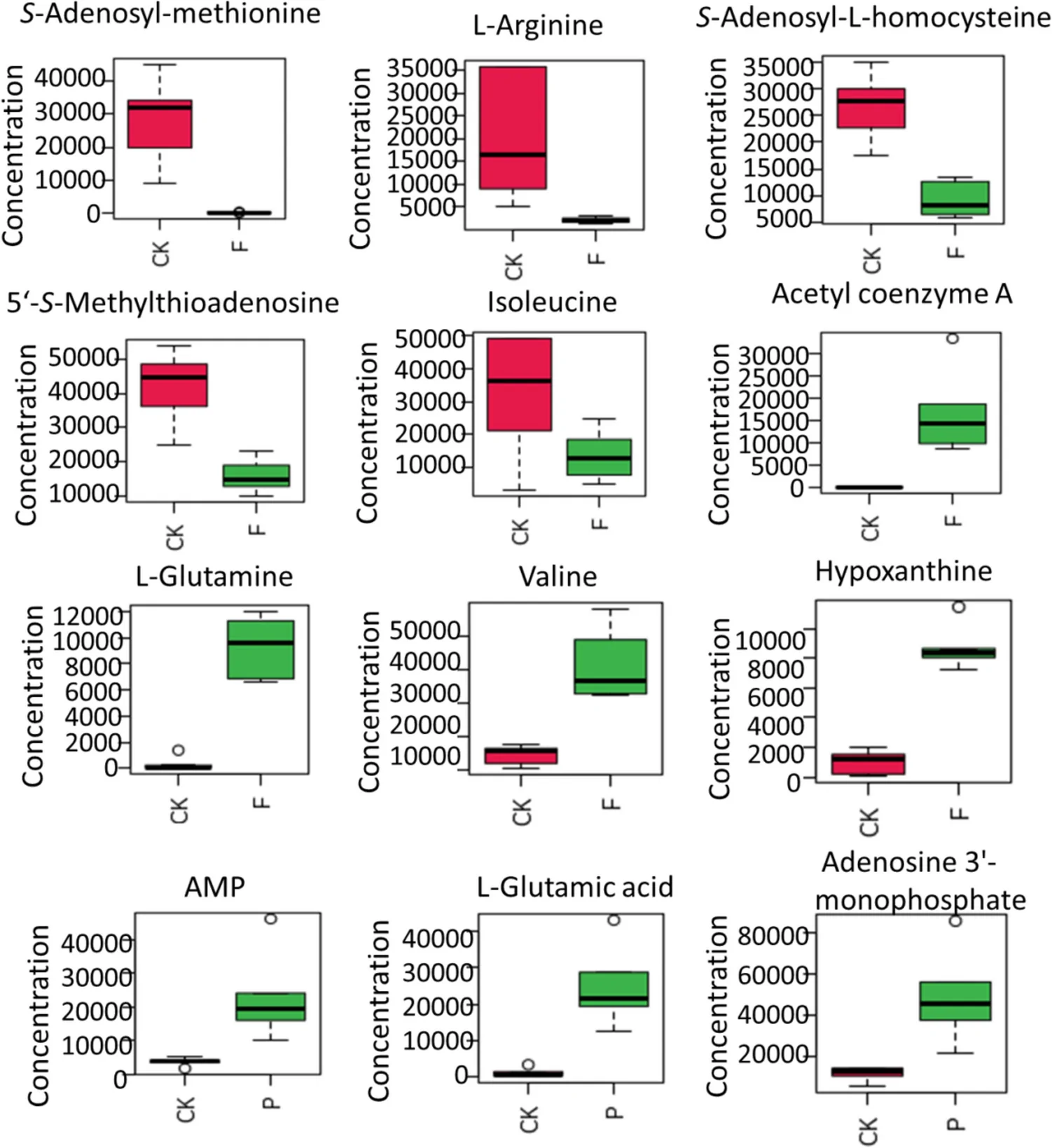
Box figure of upregulation or downregulation of some significant metabolites in *S cerevisiae* cells in group F and group P compared with group CK, analyzed on 2-day cultivation. Each group contained 6 biological replicates. Statistical significance was estimated by *t* test (*P* < 0.05). The circles in the diagrams refer to outliers

Amino acids are substrates for proteins and play an important role in cell growth and proliferation, as well as being important regulators of key metabolic pathways (Liu et al. 2016). Amino acids are key primary metabolites that help yeast cells resist various stresses and maintain metabolism and energy levels (Mailloux et al. 2013; Zhang et al. 2022). FA and* p*-CA affected the metabolism of alanine, glutamate, aspartate, proline, arginine, cysteine, and methionine. FA also affected phenylalanine and pyruvate metabolism, and the degradation of valine, leucine, and isoleucine, while *p*-CA affected the metabolism of glycine, serine, threonine, glutamine, glutamic acid, and glutathione. The differential metabolites such as *S*-adenosylmethionine, L-arginine, cysteine, methionine, and L-isoleucine were significantly downregulated, and acetyl-CoA, L-glutamate, L-threonine, L-glutamine, and L-valine were upregulated considerably, as shown in Fig. 7.

Downregulation of *S*-adenosylmethionine reduces the production of methionine, a one-carbon unit, which provides the active methyl group (Chang et al. 2018). Methionine is a raw material for purine and pyrimidine synthesis and directly affects amino acid and nucleotide metabolism. Functional amino acids (arginine, leucine, proline, cysteine, glutamine, etc.) are necessary to sustain vital activity, cell growth, as well as the development of the immune system (Wu 2009). Abnormal metabolism of amino acids like arginine, glycine, proline, threonine, serine, alanine, aspartate, alanine, and glutamate can disrupt the dynamic balance of the entire cell, impairing cell growth and possibly leading to death (Orlando et al. 2008). Decreased synthesis of most amino acids led to a reduced contribution of amino acid-related metabolic pathways to protein synthesis, which disrupted most protein synthesis. Glutathione, an important free radical scavenger and antioxidant, which is an essential metabolite in the oxidative stress reaction, can reduce reactive oxygen radicals to provide antioxidant benefits (Shanker et al. 2005; Matityahu et al. 2006; Misak et al. 2018). The addition of FA and* p*-CA led to the accumulation of free radicals in yeast cells, disrupted the redox balance in cells, and led to oxidative stress. Yeast cells resist the stress of inhibitors by generating greater substantial antioxidant capacity, to reduce the damage to cells.

The metabolomic analysis revealed that glutamine, glutamic acid, and valine exhibited a significant upregulation trend. It is most probably the self-protection mechanism of responding to inhibitor stress. Glutamine is not a required amino acid, and it can be synthesized from glutamate, valine, and isoleucine and provides an essential source of nitrogen for cells (Prinsi and Espen 2015). Glutamine also has the potential to produce bases, thus reducing to some extent the damage to cells caused by acidic substances. Yeast cells under the stress of FA and* p*-CA have increased organismal demand for glutamine to minimize cell damage.

Nucleotides are the essential substances for cell growth and reproduction. It is the precursor of synthesizing biological macromolecules like DNA and RNA, and is also involved in cell metabolism. Under pressure from FA and* p*-CA, the metabolites, including L-glutamine, adenine nucleotide, hypoxanthine, adenosine 3-phosphate, and adenylate, which were related to purine metabolism, altered significantly and showed a significant upregulation trend. Purine metabolism plays a major role in tolerating stress. These differential metabolites were all involved in the de novo synthesis pathway of purine nucleotides (Boza et al. 2000), where glutamine is the raw material for synthesizing purine nucleotides. The reaction is divided into two main stages: the synthesis of hypoxanthine nucleotide (IMP) and the production of adenine nucleotide (AMP) and guanine nucleotide (GMP) from IMP.

L-Glutamine is essential for the utilization of both catabolic and anabolic processes of nitrogen and is the primary source of intracellular nitrogen (Fayyad-Kazan et al. 2016). Furthermore, the production of other amino acids and nucleotides uses glutamine as a precursor (Ding et al. 2011). Yeast cells are thought to protect other cells from FA and *p*-CA stress by producing additional precursor chemicals. Hypoxanthine and adenosine 3-phosphate are intermediates in the de novo synthesis of purine nucleotides (Dewulf et al. 2022). With the increase of synthetic raw material glutamine, these two substances also accumulate in yeast cells. It is speculated that they may have great help in improving cell tolerance. Lipids are a large group of organic compounds that are indispensable for maintaining normal life activities of organisms, and are the storage and structural substances of biological cells (Gao et al. 2019). The most significant differential metabolites affecting fatty acid degradation in this study were palmitic acid and acetyl-CoA, which showed a significant upregulated trend. The final product of palmitic acid β-oxidation is acetyl-CoA, which requires seven rounds of β-oxidation, consumes two high-energy phosphate bonds, and generates a large amount of energy, which is an essential source of intracellular energy (Adeva-Andany et al. 2019). Acetyl-CoA then enters the TCA cycle and is completely oxidized to carbon dioxide and water. The TCA cycle is essential to the energy acquisition of organisms and is a common pathway for the complete oxidative decomposition of sugars, proteins, and fats (Hou et al. 2021). Due to the upregulation of palmitic acid and acetyl-CoA, the energy obtained by the cell from oxidizing sugar or other substances increases (Shi and Tu 2015), which is a self-protection of yeast cells against the stress of inhibitors to generate more energy.

In this study, the inhibitory mechanism of FA and *p*-CA on *S. cerevisiae* was investigated by SEM and LC–MS. The results showed that FA and *p*-CA interfered with the function of the cell wall and of the cell membrane of yeast cells, and the changes of differential metabolites destroyed the metabolic balance of the whole cell, blocked the synthesis of some proteins, and then affected the growth of cells. However, yeast cells respond to FA and *p*-CA inhibition by forming metabolic networks through metabolic pathways. The results can help us analyze the role of very different metabolites in the process of phenolic stress.

## Data Availability

Data will be made available on request.
